# New methods to analyse microarray data that partially lack a reference signal

**DOI:** 10.1186/1471-2164-10-522

**Published:** 2009-11-13

**Authors:** Neeltje Carpaij, Ad C Fluit, Jodi A Lindsay, Marc JM Bonten, Rob JL Willems

**Affiliations:** 1 Department of Medical Microbiology, University Medical Centre Utrecht, room G04.614 PO BOX 85500, 3508 GA Utrecht, The Netherlands; 2 Department of Cellular and Molecular Medicine, St. George's, University of London, London, UK

## Abstract

**Background:**

Microarray-based Comparative Genomic Hybridisation (CGH) has been used to assess genetic variability between bacterial strains. Crucial for interpretation of microarray data is the availability of a reference to compare signal intensities to reliably determine presence or divergence each DNA fragment. However, the production of a good reference becomes unfeasible when microarrays are based on pan-genomes.

When only a single strain is used as a reference for a multistrain array, the accessory gene pool will be partially represented by reference DNA, although these genes represent the genomic repertoire that can explain differences in virulence, pathogenicity or transmissibility between strains. The lack of a reference makes interpretation of the data for these genes difficult and, if the test signal is low, they are often deleted from the analysis. We aimed to develop novel methods to determine the presence or divergence of genes in a *Staphylococcus aureus *multistrain PCR product microarray-based CGH approach for which reference DNA was not available for some probes.

**Results:**

In this study we have developed 6 new methods to predict divergence and presence of all genes spotted on a multistrain *Staphylococcus aureus *DNA microarray, published previously, including those gene spots that lack reference signals. When considering specificity and PPV (i.e. the false-positive rate) as the most important criteria for evaluating these methods, the method that defined gene presence based on a signal at least twice as high as the background and higher than the reference signal (method 4) had the best test characteristics. For this method specificity was 100% and 82% for MRSA252 (compared to the GACK method) and all spots (compared to sequence data), respectively, and PPV were 100% and 76% for MRSA252 (compared to the GACK method) and all spots (compared to sequence data), respectively.

**Conclusion:**

A definition of gene presence based on signal at least twice as high as the background and higher than the reference signal (method 4) had the best test characteristics, allowing the analysis of 6-17% more of the genes not present in the reference strain. This method is recommended to analyse microarray data that partially lack a reference signal.

## Background

Comparative Genomic Hybridisation (CGH) microarray studies are applied to identify genetic diversity in both eukaryotes and prokaryotes [1-8]. In bacteria microarray-based CGH has been used in genome typing and comparative phylogenomic analyses to assess genomic regions or genes involved in bacterial adaptation [9].

The relationship between the intensity of a hybridised probe and the presence or divergence of a gene is crucial in microarray-based CGH [10]. Features such as secondary structure, melting temperature, and even target characteristics make it difficult to define a cut-off intensity for gene presence [11,12]. In general, DNA from a test strain is co-hybridised with differently labelled DNA from a reference strain in order to sidestep these issues in microarray analysis. The use of a reference allows a comparison of signal intensities and the determination, for each DNA fragment in the reference strain, whether it is present or divergent in the test strain [13]. The reference strain serves also as quality control for spots on microarray slides. In principle all spots should yield a signal for the reference, as it contains all genes. When a spot does not yield a signal with the DNA of both the reference strain and the test strain, the spot will be deleted from the analysis. The production of a good reference becomes more difficult or even unfeasible when the probes present on the microarray are not based on a single strain, but represent multiple genomes or even the pan-genome of a species. It is to be expected that the number of pan-genome arrays built from multiple strains will only increase with the rapid expansion of available (bacterial) whole genome sequences [9].

There are already several methods to analyse microarrays, which partly lack a reference [8,14-16]. However, in these approaches spots without reference and lacking a test signal are flagged as poorly performing, and removed from the analysis. Consequently, these genes cannot be classified with certainty as present or divergent. In this study we developed novel methods to determine the presence or divergence of all genes in a *Staphylococcus aureus *multistrain PCR product microarray-based CGH approach, including those that lack a reference signal by using performance data from all the spots on the microarray.

## Methods

### Description of the DNA microarray and its use

All laboratory protocols have been described in detail by Witney et al. [16] and are registered at BμG@Sbase http://bugs.sgul.ac.uk/E-BUGS-30.

The *S. aureus *DNA microarray used in this study, which consists of PCR-based probes for all open reading frames (ORFs) of seven *S. aureus *strains, has been described and validated previously and is summarised here [16]. In short, all ORFs of MRSA252, which served as base strain, were added to the microarray design followed by the addition of probes for genes from the other strains that are absent in, or show significant divergence from the genes of MRSA252 based on BLAST bit scores. The order in which the probes for the genes of the strains were added to the array was: MRSA252 (base strain), N315, Mu50, COL, NCTC8325, MW2, and MSSA476 [16]. In total, the microarray consisted of 3623 PCR products spotted in duplicate representing every predicted open reading frame of the seven strains [8,16]. Around 75% of the PCR products (n = 5478 in duplo) represent MRSA252, while around 25% of the PCR products (n = 1768 in duplo) were obtained from the other six strains.

All strains were cultured on tryptic soy agar sheep blood plates at 37°C overnight. DNA of the reference strain and the test strains was isolated using the QIAGEN genomic-tip 100/G column and an Edge Biosystems Bactererial Genomic DNA purification kit (Edge Biosystems, Gateshead, United Kingdom).

DNA of all seven sequenced *S. aureus *strains, labelled with Cy3, was hybridised in duplicate on an array, with DNA of MRSA252, which was labelled with Cy5 as reference signal. Labeling was performed as described previously [8,16].

Microarray images were quantified with ImaGene software (Biodiscovery, http://www.biodiscovery.com, El Segundo, California, United States). The two pictures per slide, one for every dye, were analysed separately in ImaGene. Fully annotated microarray data are deposited in BμG@Sbase http://bugs.sgul.ac.uk/E-BUGS-30 and have been retrieved for this study.

The background was calculated automatically and separately per colour and spot by ImaGene software.

### Data-processing

Since a reference signal was available for 75% of the microarray spots, only for this part hybridisation data could be analysed using ratios of test, Cy3, and reference, Cy5, signal. For this purpose the data were dissected into two different data sets. The first data set consisted of the hybridisation results of the MRSA252 specific spots (75% of the spots in duplicate) containing a reference signal (further indicated as MRSA252 spots). Hybridisation signals for these MRSA252 spots were analysed using GACK (further referred to as GACK method), which is a well-documented standard analysis method [4,17,18] and also the new analysis methods developed in this study. The second data set that includes all spots, i.e. both the non-MRSA252 spots that lack a reference signal and the MRSA252 spots, were only analysed using the new developed methods. The MRSA252 spots were also analysed with the new analysis methods to compare the outcome of the methods with that of the GACK method.

**Figure 1:**
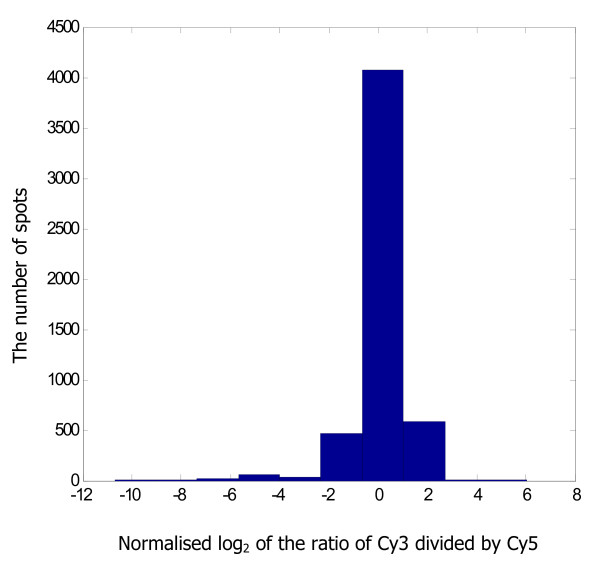
**Distribution of the normalised ratio**. Example of a histogram of one slide constructed using Matlab2006b showing the distribution of the normalised signal intensities. Only the MRSA252 spots were taken into account in the number of spots and the flagged spots were filtered.

In a first step only the MRSA252 spots on all slides were analysed. For every array the bad spots were filtered. Filtering was applied to exclude spots for which the reference, Cy5, signal was less than two times the background value of that particular spot. Also the spots, which ImaGene automatically defines as bad or empty spots, were excluded from the data. Slides were then normalised per array to correct for differences in labelling-efficiency, hybridisation, scanning conditions, and slide quality. For every particular spot the intensity of Cy3 minus the Cy3 background signal and Cy5 minus the Cy5 background signal was calculated, after which Cy3 to Cy5 ratios were calculated and log_2_-transformed. Per array the median and standard deviation of all ratios were calculated. The arrays were then subsequently normalised per spot, by first subtracting the median ratio of that particular array followed by dividing the resulting ratios per spot by the specific standard deviation of all ratios for that array. This specific normalisation is also called auto-scale normalisation. The estimated probability of presence of each gene or DNA fragment was determined using a GACK-transformation http://falkow.stanford.edu/whatwedo/software/software.html[19]. Using GACK transformation, it is possible to dynamically choose cut-offs for determining presence or divergence of genes or DNA fragments based on the shape of the distribution. For this study data were transformed to binary output using a threshold of 50% estimated probability of being present (EPP) in order to call genes present or divergent. To use GACK the data need to be normally distributed, which was the case in this study (Figure 1).

To analyse the second data set, which include the non-MRSA252 as well as the MRSA252 spots, all slides were again normalised separately. This means that first the intensities of Cy3 minus the Cy3 background signal for all spots and Cy5 minus the Cy5 background Cy5 signal for the MRSA252 spots were calculated. Because of the lack of a reference signal, the non-MRSA252 spots could not be normalised in the ratio dependent way or by auto-scale normalisation since the latter will reduce differences in the intensities, which compromises calculation of an accurate cut-off for gene presence or divergence. For this reason the second data set was analysed using different newly developed methods (see below). Filtering of the MRSA252 spots in this data set was performed identical as in the first data set; by excluding the automatically flagged spots by ImaGene and spots with a Cy5 signal intensity, which is less than two times the Cy5 background signal.

The results of the new analysis methods were validated by comparing the hybridisation data of the seven sequenced strains used in the array with the predicted presence and divergence of genes based on the GACK method and on the genome sequence data. Performance of the new method was determined by calculating sensitivity, specificity, PPV and NPV of the new methods. Sensitivity is calculated by dividing the number of genes predicted to be present in the control strains based on the new analysis method, by the number of genes that are considered to be present based on the annotation of Witney et al [16]. The calculated sensitivity of an analysis method was only based on the extra spots for each strain that were added to the array. For MRSA252 this means around 75% of the total of all spots on the array, since this strain was added first on the array. So, if for instance only 148 of in total 176 NCTC8325-specific sequences were identified as being present by an analysis method, the sensitivity of that analysis method would be 84.09%.

Specificity is the proportion of the divergent sequences, which are correctly identified as divergent sequences in an analysis method. This means that for, e.g., strain NCTC8325 all 170 probes spotted extra for strains MW2 and MSSA476 should be divergent in the NCTC8325 genes, since MW2 and MSSA476 were added after NCTC8325. If, however, in this case only 119 sequences were called divergent by an analysis method, the specificity would be 70%.

The positive predictive value (PPV) is the number of the true positive sequences divided by the total number of sequences that were indicated as positive (true and false-positive) in the different analysis methods. This means that if 104 of the 5484 probes that are called present for MRSA252 by an analysis method are false-positive, the PPV would be 98.10% (5380/5484)

The negative predictive value (NPV) is the number true divergent sequences divided by the total number of sequences indicated as divergent. So, in the case that only 902 of 922 sequences called divergent by an analysis method are true divergent, the NPV would be 97.83% (902/922).

For the MRSA252 spots, sensitivity, specificity, positive predictive value (PPV) and negative predictive value (NPV) of the new methods were calculated and compared with the values obtained with the GACK method.

In total six different approaches were used to analyse the data. In the first method a cut-off per array and in the other five methods a cut-off per spot was calculated.

### Cut-off per array

To calculate the cut-off per array, log_2 _values of the intensities of the reference MRSA252, Cy5 signal, on the MRSA252 and the non-MRSA252 spots were determined. The cut-off is that intensity value where 95% of the MRSA252 spots will be called positive and 95% of the non-MRSA252 spots negative. Only the intensities of the MRSA252 reference signal were included because for this strain it is exactly known which sequences should be present and which diverge; all MRSA252 probes have to be present and all non-MRSA252 probes have to be divergent.

### Cut-off per spot

Five different approaches were used to determine the cut-off value per spot for presence and divergence of the sequences, which are explained below. All approaches analysed the hybridisation signals for each spot separately and generated a cut-off per spot to determine whether a sequence was conserved or divergent. With the consequence that intensity above the cut-off means that a gene is present. Using these cut-offs, sensitivities and specificities, PPV and NPV were calculated as described above.

#### 1. Cut-off based on two times the background

In the first approach non-MRSA252 sequences were considered present when the value of the Cy3 intensity of a particular spot was higher than twice the background signal of that spot. In short: gene is present when Cy3 intensity > 2 × Cy3 background signal.

#### 2. Cut-off based on reference signal intensities

In the second approach the non-MRSA252 sequences were considered present when the value of the Cy3 intensity of a particular spot was higher than the Cy5 signal of that spot. Cy5 signals for non-MRSA252 spots are considered background noise in this approach, caused by, among other things, cross-hybridisation. So, in this second method, we hypothesized that signal intensity of the test strains for conserved sequences has to be at least above the value of the reference signal. In short: A gene is present when Cy3 intensity > Cy5 intensity.

#### 3. Cut-off based on the minimal ratio of positivity

The first step in this third approach is to determine per array the lowest test/reference (Cy3/Cy5) intensity ratio for the MRSA252 spots, thus for spots for which a reference signal is available, and which were predicted to be positive based on GACK analysis. Subsequently the reference intensity of every individual spot was multiplied with the value of this lowest Cy3/Cy5 ratio. This way the lowest Cy3 value per spot predicted to be present was calculated. The last step in the calculation is that Cy3 intensities, which were greater than the Cy5 value of that spot multiplied by the lowest Cy3/Cy5 ratio, were considered to represent sequence presence. In short: A gene is present when Cy3 intensity > minimal ratio × Cy5 intensity.

#### 4. Cut-off based on two times the background and reference signal intensities

This approach consists of a combined calculation of the first and second approach (see above). In this method sequences were considered present when the value of the Cy3 intensity of a particular spot was higher than twice the background signal and when the Cy3 intensity was higher than the Cy5 intensity of that spot. In short: A gene is present when Cy3 intensity > (2 × Cy3 background signal) and > Cy5 intensity.

#### 5. Cut-off based on two times the background and the minimal ratio of positivity

This last approach is a combined calculation of the first and the third approach. The first step in the last approach is to determine per array the lowest Cy3/Cy5 ratio for MRSA252 spots, as described above. Cy3 intensities, that were twice the background and greater than the Cy5 value of that spot multiplied by the lowest Cy3/Cy5 ratio were considered to represent sequences presence. In short: A gene is present when Cy3 intensity > (2 × Cy3 background signal) and > (minimal ratio × Cy5 intensity).

## Results

Fully annotated microarray data have been deposited in BuG@Sbase (accession number E-BUGS-85; http://bugs.sgul.ac.uk/E-BUGS-85) and also ArrayExpress (accession number E-BUGS-85). From the fourteen control arrays, three (hybridised with MRSA252 (n = 1) and MSSA476 (n = 2)) were excluded from further analysis, because of poor hybridisations with low signal intensities for the test strain.

### Cut-off per array

**Figure 2:**
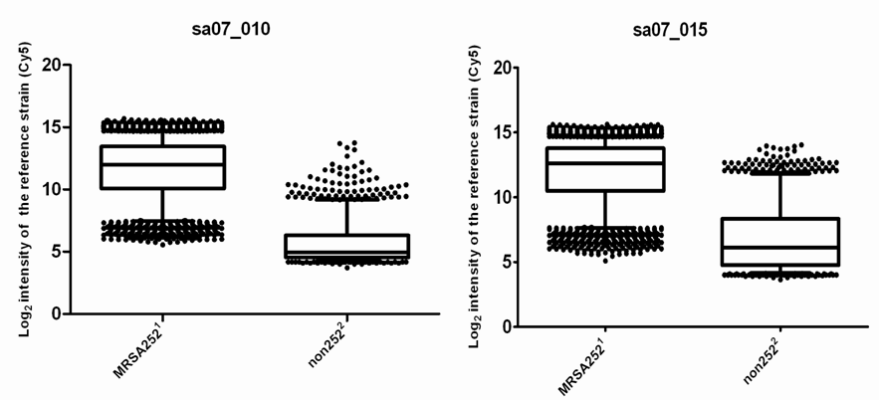
**Box plots for determining cut-offs for the presence or divergence of genes**. Example of two different box plots of two different microarray slides (Sa07_010 and Sa07_015) constructed using GraphPad Prism5. ^1^MRSA252 are the spots originating from MRSA252 and should give a signal for in the Cy5 dye; ^2 ^Non252 are the spots originating from the other six strains (N315, MW2, Mu50, NCTC8325, COL and MSSA476), which are absent in the MRSA252 and so should not yield a Cy5 signal. The box plots illustrate the log_2 _of the raw intensity of the reference (Cy5) channel for the MRSA252 and the non-MRSA252 spots separately. The horizontal line in the box denotes the median of the intensity. The log_2 _of the signal for 50% of the spots falls within the boxes and the dots account for the upper and lower 5% of the spots. These pictures clearly show that height of the raw intensity does not correlate with the presence or divergence of a gene. These box plots indicate highly variable intensities between different spots on the same microarray slide.

Box plots, in which the log_2 _values of the intensities of the reference MRSA252 (Cy5) signal for the MRSA252 and the non-MRSA252 spots are presented, indicate highly variable intensities between different spots on the same microarray slide (Figure 2). Based on these results we concluded that a single cut-off per array was not feasible and alternative methods were developed.

### Cut-off per spot

Sensitivities, specificities, PPVs and NPVs of the different approaches are described in Table 1. Specificities for all spots ranged from 7.85% for method 3 (cut-off based on the minimal ratio of positivity) to 81.61% for method 4 (cut-off based on two times the background and reference signal intensities). Method 4 also had the highest PPV (76.13%) for the second data set, including all spots. Method 3 showed for the MRSA252 data set nearly the same values as the GACK analysis (less than 2% probes were misclassified in method 3 compared with the GACK analysis).

**Table 1:** Sensitivity, specificity, PPV and NPV of the newly developed analysis methods based on calculated cut-offs per spot.

Analysis method	Test characteristics	MRSA252^a^	All spots^b^
1 Cut-off based on two times the background	Sensitivity^c^	96.90%	86.40%
	Specificity	15.21%	76.68%
	PPV	89.43%	73.64%
	NPV	39.91%	82.34%

2 Cut-off based on reference signal intensities	Sensitivity	50.31%	90.06%
	Specificity	100%	19.79%
	PPV	100%	51.96%
	NPV	21.38%	71.69%

3 Cut-off based on the minimal ratio positivity	Sensitivity	99.98%	98.24%
	Specificity	98.81%	7.85%
	PPV	99.84%	50.86%
	NPV	99.89%	80.90%

4 Cut-off based on two times the background and reference signal intensities	Sensitivity	50.31%	79.15%
	Specificity	100%	81.61%
	PPV	100%	76.13%
	NPV	21.38%	76.41%

5 Cut-off based on two times the background and the minimal ratio of positivity	Sensitivity	96.90%	86.24%
	Specificity	99.24%	77.88%
	PPV	99.89%	74.09%
	NPV	81.24%	82.34%

^a ^Spots based on MRSA252 ORFs (75% of all spots on the array). Flagged spots of the MRSA252 data set were filtered from the calculations.

To validate the results of the different new analysis methods the MRSA252 spots were also analysed with the new methods and compared with the results obtained with the GACK method and sensitivity, specificity, positive predictive value (PPV) and negative predictive value (NPV) of the new methods were calculated.

^b ^All spots representing genes present in MRSA252, N315, MW2, Mu50, NCTC8325, COL and MSSA476.

^c ^To calculate sensitivity, specificity, PPV and NPV of the new methods, the hybridisation results of six sequenced strains used in the array design were included with the exception of the results of MSSA476. The calculated sensitivity of an analysis method was only based on the specific spots for each strain that were added to the array. So only the MRSA252 spots were taken into account for the sensitivity for the MRSA252 strain. Specificity for a strain could only be calculated based on the strains that were added on the array after the specific strain. These genes have to be divergent, since they were not present in the specific strain. This means that for strain NCTC8325 all 170 genes spotted extra for strains MW2 and MSSA476 should be divergent in the NCTC8325 hybridisations, since MW2 and MSSA476 were added after NCTC8325.

As compared to method 4, methods 2 (cut-off based on reference signal intensities) and 3, exhibited much lower specificities (81.61% for method 4 and 19.79% and 7.85% for methods 2 and 3, respectively). In method 2 the non-MRSA252 spots were considered present when the value of the Cy3 test signal of a particular spot was higher than the Cy5 signal of that spot. However, Cy5 signals for the non-MRSA252 spots are extremely low and considered background noise, yielding a low cut-off for Cy3 test signals and a relatively high degree of false-positive results. Method 3 (cut-off based on the minimal ratio of positivity) had the best sensitivity and specificity for the MRSA252 spots, but a low specificity for the non-MRSA252 spots, from which we conclude that the Cy5 intensity, which is the only difference between the MRSA252 spots and the non-MRSA252 spots, is much lower for the non-MRSA252 spots than for the MRSA252 spots. This will give a very low cut-off, resulting in a low specificity.

Method 5 (cut-off based on two times the background and the minimal ratio of positivity) had a lower specificity and PPV (i.e. higher false-positive values) than method 4, which can be explained by the fact that the Cy3/Cy5 ratio, as used in method 3 (see above), results in too low cut-off values, and, thus, a higher degree of false-positive results.

## Discussion

The calculations for cut-off per spot described in this study provide useful tools for data analysis of microarrays that partially lack reference signals. For each of these methods, however, conclusions about negative spots should be drawn with considerable care. In the absence of a good reference, a negative test signal can mean that a particular gene is truly divergent or that the spot has been badly manufactured. For this reason we only considered specificity and PPV as important values.

While there is a wealth of approaches to analyse microarray data with a reference [10,16,20-24], few methods are available for the analysis of data from dual labelled slides that are partly without a reference [14,16,20,25,26]. The most common analysis for non-Affymetrics arrays (partly) without reference includes the use of one external reference and one cut-off for all arrays [14,20,25,26] However, such an approach appeared not optimal in this study as the variation in signal intensities was too large between and within arrays (Figure 2). The nature of the probes probably explains the large degree of variation in signal intensities, because these consisted of PCR fragments with variable length (between the 100 and 800 base pairs) [16], which were spotted with variable densities.

Genes that share considerable sequence similarity, thus scored presence based on the microarray hybridisation, but were considered divergent (based on BLAST bit scores) [16] can also explain the observed low specificities and low PPVs for the second data set (including all the spots). The BLAST bit score was used as a quality measure for gene divergence, because it reflects the length as well as the degree of sequence similarity. Although this gives in general a reliable prediction of gene presence or divergence, it is possible that two genes with significant stretches of sequence similarity will be classified as divergent based on bit-scores, e.g., when two genes differ in size due to differences in repeat numbers. Additionally, gene redundancy may also explain low PPVs. The *S. aureus *genome has numerous well-documented examples of multiple genes that show a significant level of sequence similarity, which can give false-positive hybridisation results.

Interpreting presence or divergence of genes using a multistrain microarray in the absence of a reference for all microarray spots is highly complex as illustrated in this study. This is especially true when hybridisation signal intensities fall into the marginal zone between clearly present or clearly divergent. The most important advantage, of the newly developed methods, especially methods 4 and 5 (cut-off based on two times the background and reference signal intensities and based on two times the background and the minimal ratio of positivity, respectively) over previously published methods is that they predict divergence and presence of all genes spotted on a microarray within a reasonable certainty, including the spots that lack a reference and test signal. The fact that spots, lacking a test and reference signal, are valued as poorly performing, instead of potentially representing divergent genes explains why previously reported specificities and sensitivities were slightly higher than the ones we calculate in the current study[16]. Analysis of all differentially present genes is of utmost importance, since these genes denote the accessory genome, which most likely represents the genomic repertoire that explains for a large part observed virulence, pathogenicity or transmission differences between clones.

## Conclusion

When considering specificity and PPV (i.e. the false-positive rate) as the most important criteria for evaluating new approaches for analysing microarrays that partially lack reference signals, a definition of sequence presence based on a signal higher at least twice as high as the background and higher than the reference signal (method 4) showed the best test characteristics. For this method specificity was 100% and 82% for MRSA252 and all spots, respectively, and PPV were 100% and 76% for MRSA252 and all spots, respectively. For the *S. aureus *array, which was evaluated in this study, it implied that we are now able to analyse 6-17% more of the genes not present in the reference strain than in previous publications using the same array.

## Authors' contributions

NC carried out the analysis and drafted the manuscript. AF and RW contributed in discussions and analysis. AF, RW, JL, MB helped to draft the manuscript. All authors read and approved the final manuscript.
